# Clinical Characteristics, Laboratory Parameters, and Molecular Epidemiology of Neuroinvasive Flavivirus Infections in a Hotspot Region of Eastern Croatia

**DOI:** 10.3390/pathogens14010069

**Published:** 2025-01-14

**Authors:** Dario Sabadi, Kristian Bodulić, Vladimir Savić, Nika Vlahović Vlašić, Maja Bogdanić, Ljiljana Perić, Irena Tabain, Dubravka Lišnjić, Mario Duvnjak, Snježana Židovec-Lepej, Barbara Grubišić, Ilija Rubil, Ljubo Barbić, Luka Švitek, Vladimir Stevanović, Petra Smajić, Bernarda Berišić, Mihaela Zlosa, Ivana Rončević, Tatjana Vilibić-Čavlek

**Affiliations:** 1 Clinic for Infectious Diseases, University Hospital Centre Osijek, 31000 Osijek, Croatia; dariocroatia@gmail.com (D.S.); vlahovic30@gmail.com (N.V.V.); mario.duvnjak@kbco.hr (M.D.); grubisic.barbara@gmail.com (B.G.); ilija.rubil@gmail.com (I.R.); smajicpetra@gmail.com (P.S.); bernarda.berisic@gmail.com (B.B.); mihaelazlosa@gmail.com (M.Z.); 2 Department of Infectology and Dermatovenerology, Faculty of Medicine, Josip Juraj Strossmayer University of Osijek, 31000 Osijek, Croatia; ljiperic@mefos.hr (L.P.); dubravka.lisnjic@gmail.com (D.L.); 3 Faculty of Dental Medicine and Health, Josip Juraj Strossmayer University of Osijek, 31000 Osijek, Croatia; 4 Research Department, University Hospital for Infectious Diseases “Dr. Fran Mihaljević”, 10000 Zagreb, Croatia; kristian.bodulic@gmail.com; 5 Poultry Center, Croatian Veterinary Institute, 10000 Zagreb, Croatia; roncevic@veinst.hr; 6 Department of Virology, Croatian Institute of Public Health, 10000 Zagreb, Croatia; maja.bogdanic@hzjz.hr (M.B.); irena.tabain@hzjz.hr (I.T.); 7 School of Medicine, University of Zagreb, 10000 Zagreb, Croatia; 8 Department of Immunological and Molecular Diagnostics, University Hospital for Infectious Diseases “Dr. Fran Mihaljević”, 10000 Zagreb, Croatia; szidovec@gmail.com; 9 Department of Microbiology and Infectious Diseases with Clinic, Faculty of Veterinary Medicine, University of Zagreb, 10000 Zagreb, Croatia; ljubo.barbic@vef.hr (L.B.); vladostevanovic@gmail.com (V.S.)

**Keywords:** tick-borne encephalitis, West Nile virus, neuroinvasive disease, clinical characteristics, laboratory parameters, Croatia

## Abstract

Neuroinvasive flaviviruses such as tick-borne encephalitis virus (TBEV) and West Nile virus (WNV) are widely distributed in continental Croatian regions. We analyzed clinical characteristics, laboratory parameters, and molecular epidemiology of neuroinvasive flavivirus infections in eastern Croatia. A total of 43 patients with confirmed flavivirus infection hospitalized from 2017 to 2023 were included in the study. Reverse-transcription polymerase chain reaction (RT-qPCR) was used to detect flavivirus RNA in clinical samples (cerebrospinal fluid; CSF, urine). ELISA was used for IgM and IgG antibody detection in serum and CSF with confirmation of cross-reactive samples by virus neutralization test. WNV was detected more frequently (74.4%) than TBEV (25.6%). A statistically significant age difference was found between WNV patients (median 65 years) and TBEV patients (median 36 years). Comorbidities were more frequently detected in WNV patients (hypertension 56.3 vs. 18.2%; diabetes 31.3 vs. 0%). Meningitis was the most common clinical presentation in both TBE and WNV neuroinvasive disease (WNND; 63.6 and 59.4%, respectively). In addition, some rare clinical presentations of WNND were also detected (cerebellitis, polyradiculoneuritis). No significant differences in the frequency of clinical symptoms were observed between WNV and TBEV-infected patients (fever 93.7 vs. 100%; malaise 78.1 vs. 100%; headache 75.0 vs. 100%; nausea 50.0 vs. 63.6%; vomiting 34.4 vs. 54.6%). Comparative analysis of total and differential leukocyte blood count showed similar results. However, CSF pleocytosis was higher in TBE patients, with a significant difference in the neutrophil and lymphocyte count (WNND median 48.5% and 51.5%; TBE median 10.0 and 90.0%, respectively). The length of hospital stay was 12 days for WNND and 9 days for TBE. Phylogenetic analysis of detected WNV strains revealed the presence of WNV lineage 2 in eastern Croatia.

## 1. Introduction

Flaviviruses are a group of RNA viruses transmitted primarily by mosquitoes and ticks, with some species causing diseases in humans. Neuroinvasive flaviviruses such as tick-borne encephalitis virus (TBEV) and West Nile virus (WNV) represent an important public health problem in Europe [1].

In a natural cycle, WNV circulates between birds (reservoirs) and mosquitoes (vectors) [2]. Humans and horses can become infected by mosquito bites and are considered incidental or dead-end hosts [3]. However, occasionally transmission may occur through blood transfusion or organ transplantation [2,4]. Almost 80% of WNV infections are asymptomatic, while approximately 20% of cases present with mild disease, WNV fever (WNF). Less than 1% of infected individuals, primarily the elderly, immunocompromised, or those with pre-existing medical conditions, develop WNV neuroinvasive disease (WNND), including encephalitis, meningitis, acute flaccid paralysis, or polyradiculoneuritis [1,5]. WNV meningitis typically manifests with fever, headache, nausea, vomiting, and nuchal rigidity. WNV encephalitis is a more severe form of the disease and includes symptoms such as fever, altered mental status, convulsions, focal neurologic abnormalities, and movement disorders including tremor or parkinsonism. WNV myelitis is clinically identical to myelitis caused by poliovirus and may progress to respiratory paralysis that requires mechanical ventilation. The case fatality rate in patients with WNND is approximately 10%, while long-term neurologic sequelae are common in patients with encephalitis and myelitis [6].

WNV has been established in central Europe and the Mediterranean basin, causing small or large outbreaks [1,7,8,9]. In Croatia, the first reported human cases of WNND occurred in 2012, during an outbreak in two eastern counties [10]. Until 2018, human WNV infections have been recorded annually as seasonal outbreaks or sporadic cases in late summer and autumn [11,12]. Notably, human cases were not detected from 2019 to 2021 [13], while since 2022, human WNND infections have been re-established [13,14]. WNV lineage 1 strains have circulated in Europe, causing human outbreaks since the 1950s [15]. Since WNV lineage 2 was first detected in Hungary in 2004, it has spread across central Europe and the Mediterranean, becoming the major cause of WNV outbreaks [16,17,18].

Tick-borne encephalitis (TBE) is a significant health concern in Europe and Asia [19]. In recent years, there has been an irregular but noticeable increase in TBE incidence and an expansion of its geographical range. The primary vector for the TBEV is the *Ixodes ricinus* tick, predominantly found in Europe [20]. Human infections typically result from the bite of an infected tick. Additionally, food-borne TBE infections can occur after consuming unpasteurized goat milk [21]. The European TBE is usually a biphasic disease with a first viremic phase and a second phase characterized by recurrence of fever and symptoms of the central nervous system involvement. Among patients with neuroinvasive TBE, meningitis occurs in nearly half of the cases, encephalitis in 40%, and myelitis in 5–10% [22]. The Far-Eastern TBEV usually causes a monophasic and more severe disease. The case fatality rate is 1–2% for European and 20–40% for Far-Eastern TBEV [20].

In eastern Croatia, the incidence of TBE has also shown an upward trend, mirroring the broader geographic spread of the disease across Europe. Between 2017 and 2023, human cases were annually recorded in the continental region. TBEV infection in Croatia showed bimodal seasonality with a larger peak in early summer and a smaller peak in autumn [23]. Phylogenetic analysis of Croatian TBEV strains from one human sample, ticks, and deer spleen revealed they all belong to the European TBEV subtype and are closely related [13,24].

Eastern Croatian regions are recognized as hotspots for flavivirus infections, based on reports of human clinical cases and acute infections or seropositivity in sentinel animals. Since the first records in 2012, WNV infections in humans have been detected annually in eastern counties except for 2013 and 2015 as well as 2019–2021, when no infections were reported. Almost 50% of all human WNV infections in Croatia have been recorded in the eastern region [11,12,13,14]. In addition, acute asymptomatic infections and the highest WNV IgG seropositivity in horses were continuously observed in eastern regions bordering Hungary and Serbia [12]. This is expected, since Kopački Rit Nature Park, a specific flooded area that is an ideal location for the development of extremely large mosquito populations, is located in Osijek-Baranja County [25].

Although some recent studies described the clinical characteristics of neuroinvasive flavivirus infections [26,27,28], data on the laboratory characteristics and comparison of TBE and WNND are lacking. Given the public health impact of flaviviruses in eastern Croatia, this study aimed to analyze the clinical and laboratory characteristics and molecular epidemiology of neuroinvasive flavivirus infections in hospitalized patients in this region.

## 2. Materials and Methods

### 2.1. Patients

**Figure 1:**
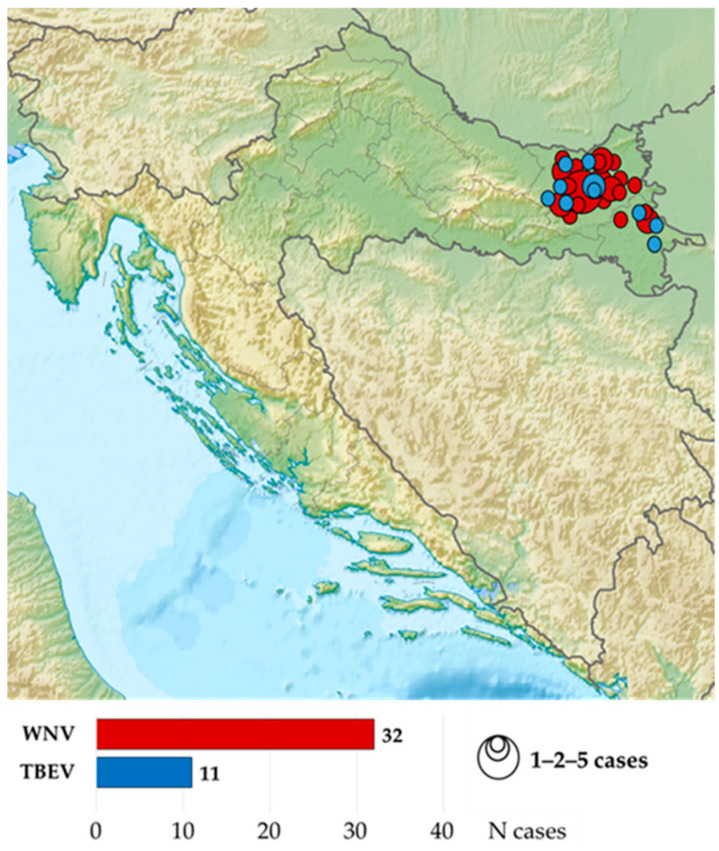
Distribution of patients with neuroinvasive flavivirus infection in eastern Croatia.

The study included 43 patients (predominantly male; *n* = 27/62.8%) with a confirmed neuroinvasive flavivirus infection hospitalized from April 2017 to October 2023 at the Department for Infectious Diseases, Clinical Hospital Center Osijek. A total of 31 patients were analyzed retrospectively from a previous cohort [11,14], while 10 patients were new ones detected during the 2023 transmission season. The patients originated from two easternmost Croatian counties (Osijek-Baranja and Vukovar-Srijem County) (Figure 1).

The median age of participants was 58 years, ranging from 6 to 89 years. The largest age group comprised individuals aged 66 to 80 (30.2%). Most patients were diagnosed with WNND (74.4%), while those with TBE accounted for 25.6%. The median duration of symptoms before hospitalization was 5 days (range 1–15), and the median length of hospital stay was 10 days (range 2–21 days). One patient with TBE was vaccinated against TBEV.

### 2.2. Methods

Data on demographics (age, sex), clinical characteristics (clinical diagnosis, symptoms, comorbidities, neurological status), and laboratory parameters in blood: total and differential white blood cell (WBC) count, C-reactive protein (CRP), erythrocyte sedimentation rate (ESR) and cerebrospinal fluid (CSF): number of cells, differential cell count, and protein levels were collected from medical records.

All patients were classified as confirmed cases according to the clinical and laboratory criteria of the European Centre for Disease Prevention and Control (EU Case definition) that include (a) virus isolation; (b) viral RNA in blood and/or CSF; (c) specific IgM antibodies in CSF; and/or (d) high IgM titer and IgG detection confirmed by a virus neutralization test (VNT) [29]. Since several recently published articles indicated the diagnostic value of urine reverse-transcription polymerase chain reaction (RT-qPCR) for confirmation of WNND and showed its superiority compared to CSF testing [30,31], urine was also tested.

#### 2.2.1. Flavivirus RNA Detection and Genotyping

TBEV and WNV RNA were detected in the CSF and/or urine samples using specific RT-qPCR assays according to protocols of Schwaiger and Cassinotti (2003) [32] and Tang et al. (2006) [33], respectively. Positive samples detected by RT-qPCR were further subjected to conventional RT-PCR according to the protocol of Weissenbock et al. (2002) [34]. The RT-PCR amplification products were Sanger sequenced (Humanizing Genomics, Macrogen Inc., Seoul, Korea) using the same primers. Genotyping and phylogenetic grouping of obtained sequences were based on comparison with strains retrieved from the GenBank and obtained using the BLAST algorithm (http://www.ncbi.nlm.nih.gov (accessed on 20 November 2024)). Maximum likelihood phylogenetic analysis was conducted and the evolutionary analyses were performed using MEGA11 [35].

#### 2.2.2. Flavivirus Antibody Detection

Serological testing (IgM and/or IgG antibodies) in serum and CSF samples was performed using commercial enzyme-linked immunosorbent assays (Euroimmun, Lübeck, Germany): Anti-TBE Virus ELISA (IgM)/Anti-TBE Virus ELISA 2.0 (IgG); Anti-West Nile Virus ELISA (IgM)/Anti-West Nile Virus ELISA (IgG). The results were calculated and interpreted as follows: IgM ratio < 0.8 negative, 0.8–1.1 borderline, and >1.1 positive; IgG RU/mL < 16 negative, 16–22 borderline, and >22 positive. Samples with positive TBEV and WNV IgM and IgG antibodies were tested for IgG avidity (Euroimmun, Lubeck, Germany): Avidity: Anti-TBE Virus ELISA (IgG); Avidity: Anti-West Nile Virus ELISA (IgG) to confirm acute/recent flavivirus infection. The avidity index (AI) was calculated and interpreted as follows: <40%, low (acute/recent infection); 40–60%, borderline; and >60%, high (previous exposure) [36,37]. TBEV and WNV cross-reactive serum samples were confirmed using a cell culture virus neutralization test (VNT). TBEV Ljubljana strain (provided by the European Virus Archive goes Global project) and the WNV lineage 2 strain isolated from a blackbird were used as antigens for the VNT. The virus titer (TCID_50_) was determined on day 5 after inoculation using the Reed and Muench formula. After inactivation (56 °C for 30 min), 25 μL of serial two-fold serum dilutions starting at 1:5 were prepared. A volume of 25 μL containing 100 TCID_50_ of the virus was added to each microtiter plate well and mixtures were incubated at 37 °C with CO_2_ for 1 h. In the final step, 50 μL of 2 × 10^5^ Vero E6 cells/mL in DMEM with 5% heat-inactivated fetal calf sera were added to each well. The plates were incubated at 37 °C with CO_2_ for five days. Starting from the third day of the incubation, the plates were checked for the cytopathic effect. Antibody titer ≥ 10 was considered positive [38].

#### 2.2.3. Statistical Analysis

Numerical variables were represented with medians, interquartile ranges, and ranges, while categorical variables were given as counts and percentages. All numerical variables were non-parametrically distributed, which was assessed graphically and with the Shapiro–Wilk test. Two-group comparisons between numerical variables were performed using the Mann–Whitney U test. The association between categorical variables was tested using Fisher’s exact or chi-square test, as appropriate. *P*-values were corrected for multiple comparisons using the Benjamini–Hochberg method. Patients with WNV and TBE were classified using binary logistic regression. Parameters best at separating the groups were identified by the best subset selection algorithm, using five-fold cross-validation as the selection criterion. All statistical tests were two-tailed with a significance level set to 95%. Data analysis was performed using R (version 4.4.2., R Core Team 2024, Vienna, Austria) with packages ggplot2 (version 2.3.3.) and ggpubr (version 0.4.0).

## 3. Results

### 3.1. Virology Results in Patients with Neuroinvasive Flavivirus Infections

The results of serology and molecular testing are presented in Table 1 and Table 2. TBE was confirmed in all 11 patients by the IgM detection in the CSF samples. Urine was RT-qPCR positive in one patient with severe meningoencephalomyelitis.

WNND was confirmed by IgM antibody detection in the CSF in 23 (71.8%) patients. In nine patients with negative CSF serology, WNV IgM antibodies and IgG seroconversion in paired serum samples were documented. Moreover, 8 (25.0%) of CSF and/or 18 (56.2%) urine samples tested positive for WNV RNA.

In five serum samples, cross-reactive antibodies were documented. In three samples WNV neutralizing antibodies were found, while in two patients WNV RNA was detected in the CSF or urine, indicating WNV infection (Table 2).

### 3.2. Clinical Presentations of Patients with Neuroinvasive Flavivirus Infections

Demographic and clinical characteristics of patients with flavivirus infections are presented in Table 3.

Comparing demographic characteristics, patients with WNND were predominantly male (56.3%), while TBE showed an even higher male prevalence (81.8%), but these gender differences were not statistically significant. A statistically significant difference (*p* < 0.001) was found between the age of WNV patients (median 65; range 6–89 years) and TBEV patients (median 36; range 14–58 years). TBEV was notably more common in the 31–50 age group (63.6%) compared to WNV (9.4%; *p* < 0.001). In contrast, WNND was more prevalent in the 71+ age group (34.4%), while no TBE cases were detected in this age group (*p* = 0.041).

There was no difference in the hospitalization duration between patients with TBEV (median 9, range 3–16 days) and WNV (median 12, range 2–21 days).

Meningitis was the most common clinical presentation in both TBEV (63.6%) and WNV patients (59.4%). No statistical difference was observed in the frequency of fever (100 and 93.7%, respectively, *p* = 0.395) and other clinical symptoms between groups. Comorbidities were more frequently reported in WNV patients (hypertension 56.3%; diabetes 31.3%) than in TBEV patients (hypertension 18.2%; diabetes 0%). However, when correcting for multiple testing this difference was no longer significant. Two patients with WNND died.

Some rare clinical presentations of neuroinvasive WNV infections such as cerebellitis (6-year-old female patient) and polyradiculoneuritis (21-year-old female patient) were also observed.

### 3.3. Laboratory Parameters in Patients with Neuroinvasive Flavivirus Infections

**Figure 2:**
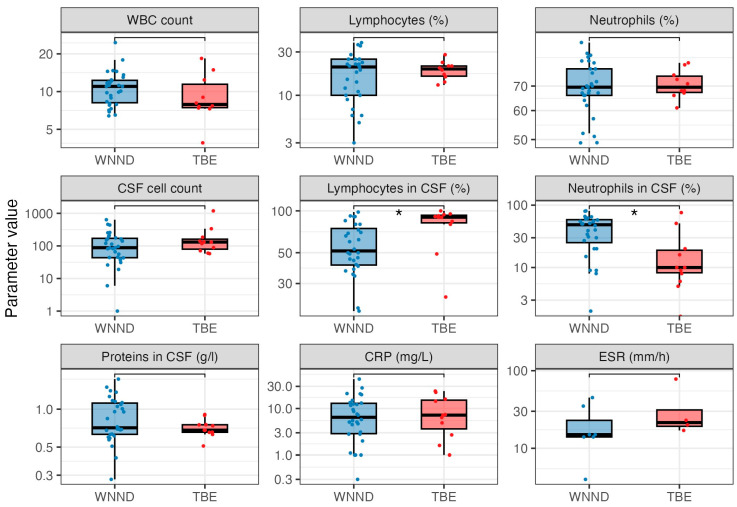
Comparison of the analyzed laboratory parameters in WNND and TBE. The boxes show the median and interquartile range of the distribution, while the whiskers extend to the minimum and maximum nonoutlier values of the distribution. Points denote individual patients. The *y*-axis is logarithmically scaled. *: *p* < 0.05 (Mann–Whitney U test, *p*-values adjusted for multiple comparisons with the Benjamini–Hochberg method). WNND = West Nile neuroinvasive disease, TBE = tick-borne encephalitis, WBC = white blood cells, CSF = cerebrospinal fluid, CRP = C-reactive protein, ESR = erythrocyte sedimentation rate.

Laboratory parameters in patients with TBE and WNND are presented in Table 4 and Figure 2.

Comparative analysis of the blood WBC count demonstrated no significant difference between the two groups. Patients with WNND had a median WBC count of 11.0 × 10^9^/L (IQR = 8.2–12.3), while TBE patients exhibited a median count of 7.9 × 10^9^/L (IQR = 7.5–11.6). The differential counts also show similar results, with lymphocyte percentages at 20.5% for WNND and 19.5% for TBE, and neutrophil percentages both at 69.5%.

CSF pleocytosis was higher in TBE patients (median 13; IQR = 80–163) compared to those with WNND (median 88; IQR = 44–171), but these differences were not significant. A notable finding was the difference in the neutrophils percentage: WNND patients had a median of 48.5% (IQR = 25.3–58.5), significantly higher than TBE patients, who had a median of 10.0% (IQR = 7.0–18.0; adjusted *p* = 0.036). Conversely, lymphocyte percentages were significantly higher in TBE (90.0%; IQR = 82.0–93.0) compared to WNND (51.5%; IQR = 40.8–74.7; adjusted *p* = 0.036).

The results of other inflammatory markers such as CRP and ESR did not differ between the groups.

A binary logistic regression was performed to understand better the relationship between the analyzed patient parameters and the clinical presentation of the disease. Parameters that best classified the patients according to disease type (TBE or WNND) were the number of comorbidities and CSF lymphocyte percentage. Increasing the number of comorbidities by one would increase the odds for patients to have WNND by 14.81 (95% CI = 2.62–217.88), *p* = 0.014. Increasing the percentage of lymphocytes in CSF by one would increase the odds for patients having TBE by 1.07 (95% CI = 1.02–1.15), *p* = 0.021 (Table 5).

### 3.4. Molecular Epidemiology of Neuroinvasive Flaviviruses

**Figure 3:**
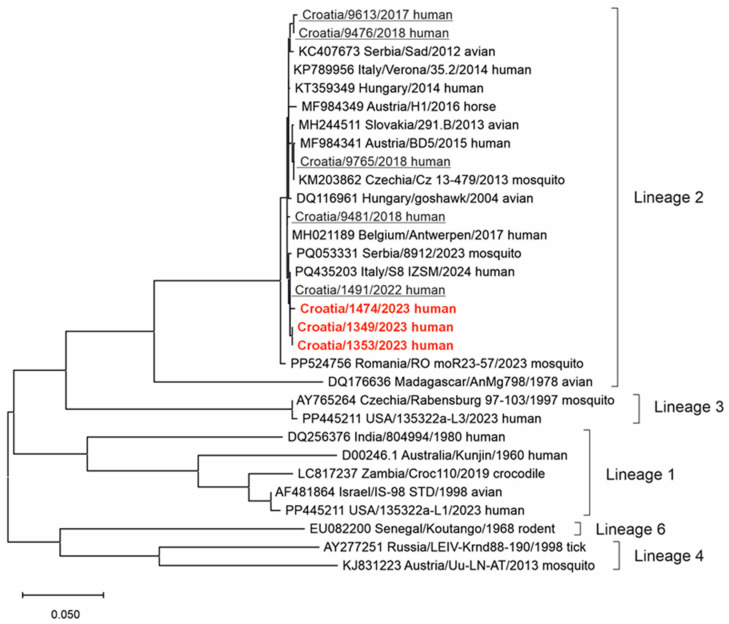
A phylogenetic tree of the West Nile virus. The evolutionary history was inferred on 794 positions of the NS5 gene. The scale bar indicates nucleotide substitutions per site. WNV isolates from eastern Croatia sequenced in previous studies [11,12,14] are underlined and those sequenced in this study are marked in red and bold. The WNV lineages proposed by Rizzoli et al. (2015) [39] are denoted on the right.

Phylogenetic analysis of detected WNV strains is presented in Figure 3. Three urine samples were sequenced in this study. In addition to that, five previously sequenced WNV isolates from eastern Croatia [11,12,14] were used for phylogenetic analysis. All eight WNV strains clustered within WNV lineage 2.

## 4. Discussion

Several previous studies analyzed the epidemiological characteristics of neuroinvasive flavivirus infections in continental Croatia, including the northwestern and northeastern regions [10,11,12,14,23]; however, data on clinical and laboratory characteristics are lacking. This study analyzed hospitalized patients from two easternmost Croatian counties recognized as hotspots for neuroinvasive flaviviruses since 2012.

WNV infections were more frequent (74.6%) than TBE (25.4%). Comparing the age of Croatian patients with WNND and TBE, significant differences in the distribution of cases were observed between the causative virus and age groups. Compared to patients with TBE (median age 36 years), the median age of patients with WNND was 65 years. While 75.0% of patients with WNND were aged 51+ years (40.6% aged 51–70 years and 34.4% aged ≥71 years), TBE patients were mostly younger than 50 years (63.6% aged 31–50 years and 18.2% ≤30 years).

Older age was recognized as a risk factor for WNND in many studies. In the wider European area, the average age among WNND cases was 64.9 years, ranging from 63.3 to 67.0 years [40], which is similar to our results. Data for TBE in European countries show that the median age of patients is 49 years [41], which is higher than in patients from eastern Croatia included in this study (36 years). However, previous national data on hospitalized TBE patients (2017–2023) showed a median of 45 (IQR = 29–59) years [23].

While the majority of TBE patients were males (81.8%, male-to-female ratio 4.5:1), WNV showed no significant gender predisposition (56.3% of patients were males and 43.7% were females; male-to-female ratio 1.3:1). Male predominance in TBE was also observed in previous Croatian studies [14,23]. A similar gender distribution of TBE patients was observed in the rest of Europe. In 2022, 20 European Union/European Economic Area (EU/EEA) countries reported a total of 3650 cases of TBE, with a higher prevalence among men (male-to-female ratio: 1.5:1) [42]. In addition, the gender distribution of WNND analyzed retrospectively in a wide European area between 2006 and 2021 was the same as in our patients (males 62.6%, females 37.4%) [40].

In our study, WNND patients presented mainly with meningitis (59.4%) and meningoencephalitis (21.9%). A similar distribution of meningitis and encephalitis was observed in TBE patients (63.6 and 27.3%, respectively). In contrast, in a European multicenter study on TBE (Austria, Czech Republic, Latvia, Lithuania, Poland, Slovenia) conducted from 2010 to 2017, 37.3% of patients presented with meningitis, 43.4 and 5.8% with moderate and severe meningoencephalitis, and 13.3% with meningoencephalomyelitis [43]. A large-scale study on hospitalized patients with WNV infection in Israel found encephalitis as the main clinical presentation in 57.9% of patients, while meningitis was detected in only 15.9% of patients [44]. A higher proportion of WNV encephalitis compared to our study was also detected in two Serbian studies conducted among patients with WNND [45,46].

Some rare clinical presentations of WNND were also detected in Croatian patients. Cerebellitis was observed in one 6-year-old child and polyradiculoneuritis in a 21-year-old patient. So far, only sporadic cases or small case series of WNV cerebellitis have been published. In 2002, a patient presenting with fever and cerebellar ataxia was reported in the USA [47]. A two-year-old patient presenting with acute profound balance and gait disturbances and intentional movement dysmetria was recorded in New Orleans [48]. In 2007, encephalitis was detected in a healthy 10-year-old patient in Omaha [49]. WNV presenting as acute cerebellar ataxia in an immunocompetent adult patient was described in 2017 in Tunisia [50]. Cerebellar dysfunction was detected in eight patients with WNND during the 2012 Serbian outbreak, with gait instability being the most prominent symptom. In addition, one patient presented with polyradiculoneuritis, which is another less common manifestation of WNV infection [46].

In patients with WNND included in our study, the most frequently detected symptoms were fever (90.6%), malaise (78.1%), and headache (75.0%). Nausea was present in 50.0%, neck stiffness in 46.9%, and vomiting in 34.4% of patients. Other less frequent symptoms were vertigo (18.8%), myalgia (18.8%), photophobia (6.3%), and arthralgia (9.4%). In a recently published US study in patients with WNND, malaise was the most frequently reported symptom (72%), followed by fever (66%), headache (55%), altered mentation (64%), diarrhea and/or vomiting (48%), myalgias (36%), and focal motor signs, (monoparesis, hemiparesis, or quadriparesis; 22%) [51]. A case series analysis of clinical characteristics in hospitalized patients with WNND in Italy showed that the most common symptoms at admission were confusion/agitation (85.7%) and postural instability/ataxia (71.4%). Fever and headache were present in 42.8% each, photophobia in 28.6%, and seizures in 57.1% of patients [52].

One recently published study found differences in the frequency of clinical symptoms in WNND in immunosuppressed and immunocompetent patients. Immunocompetent patients more frequently reported headache (63 vs. 42%) and myalgias (44 vs. 21%). In contrast, immunosuppressed patients had higher rates of altered mental status (77 vs. 57%) and myoclonus (19 vs. 4%) [51].

Comparing the Croatian WNND and TBE patients, comorbidities were more frequently detected in WNV patients (hypertension 56.3 vs. 18.2%, diabetes 31.3 vs. 0%), which is expected, since patients with WNV were older than TBEV patients (median age 65 and 36 years, respectively). In addition, according to our results, increasing the number of comorbidities by one would increase the odds for patients to have WNND by 14.81.

The observed CSF pleocytosis in our patients with WNND (median 88 cells) was within the range of the patients with WNV recorded in other countries (mean cell count 38–300/mm^3^) [53,54,55]. Similarly, the CSF pleocytosis in patients with TBE (131 cells) was similar to other reports in the same patient groups [56,57].

Analyzing the laboratory parameters in patients with WNV and TBE, no significant difference was observed in total and differential blood WBC count, ESR, and CRP. The number of WBC in the CSF was also similar (medians 88 and 131, respectively); however, the proportion of lymphocytes was significantly higher in TBE (median 90.0%) than in WNND (median 51.5%). Similar to our results (median 48.5%), the proportion of neutrophils in the CSF of WNV patients was 48.5% in a German study [58]. This proportion was significantly higher compared to TBE patients in the Croatian study (median 10.0%). Results of the binary logistic regression show that an increase in the lymphocyte percentage in CSF by one would increase the odds for patients having TBE by 1.07.

Despite well-established differences in the clinical presentation/course of WNND and TBE (biphasic disease course in European TBEV, frequency and severity of post-encephalitic sequelae, etc.), studies have shown a degree of similarity in the pathogenesis of these neuroinvasive arbovirus infection.

The most recognizable feature of WNND immunopathogenesis is extensive neuronal death caused by direct virus-mediated apoptosis and proinflammatory cytokines synthesized by microglia upon recognition of viral RNA with TLR-3-dependent mechanisms. In addition, WNND is characterized by an intensive infiltration of cytotoxic WNV-specific CD8+ T-cells recruited by chemokines synthesized mainly by virus-infected neurons [59]. Neuronal death is also considered a critical feature of TBEV infection, with recent studies providing new evidence on the molecular mechanisms responsible for this aspect of TBE pathogenesis. Fares et al. (2021) have shown that neuronal death in TBEV-infected human neuronal/glial cells involves multiple mechanisms including extrinsic apoptotic pathways as well as pyroptosis with no upregulation of major autophagic genes [60]. Future studies should also address the possible contribution of pyroptosis and autophagia to neuronal death in WNND.

Analysis of cytokines, chemokines, and growth factors in the CSF of patients with WNND and patients with European TBE in the meninogencephalitic phase of the disease has been a focus of many studies attempting to identify biomarkers of disease severity and/or outcome and find disease-specific features of their immunopathogenesis [61,62]. Our study on the cytokine composition of the CSF from WNND patients has shown increased concentrations of the key cytokines associated with innate and early acute phase responses (IL-6) and Th1-type immune responses (IFN-γ) supporting the concept of these molecules as key players in the pathogenesis of this disease. Contrary to the WNND findings, cytokine composition in the CSF of TBE patients reveals a much more complex and diverse network of early innate immune response cytokines, Th1, Th2, Th9, Th22, Th17, and anti-inflammatory cytokines that influence the pathogenesis of the disease as well as its possible outcomes [62]. Therefore, further comparative studies on well-standardized cohorts of patients with neuroinvasive arboviral diseases will be needed to identify possible biomarkers that could be clinically useful and possibly identify disease-specific patterns of biological response modifiers in TBE and WNND.

Phylogenetic analysis of detected Croatian WNV strains shows the circulation of WNV lineage 2. Two major WNV lineages (lineages 1 and 2) have played a significant role in human outbreaks in Europe in recent years [63]. A recently published article that analyzed the evolution and genetic diversity of WNV in Europe shows that WNV-2a (sub-lineage of WNV-2) has been predominant, representing 73% of all sequences collected in Europe, and has spread to at least 14 countries. WNV-1, the second-largest lineage, has been identified in seven European countries (Austria, Italy, Spain, France, Hungary, Romania, and Portugal). A small number of WNV-1 genomes have been collected from human samples in Italy and Spain [64].

A limitation of this study that needs to be addressed is the relatively small number of analyzed patients; therefore, the results should not be generalized. However, due to a lack of data, the presented results provide a better understanding of the epidemiology, clinical characteristics, and laboratory parameters in neuroinvasive flavivirus infections.

Recent global trends including climate changes, extensive urbanization, increasing traveling, and demand for transportation of goods have contributed to alterations in the distribution, spreading, and seasonality patterns of flaviviruses and their vectors, leading to the co-circulations of WNV and TBEV in certain areas, including southeastern Europe [65]. Therefore, stringent continuous epidemiological surveillance on the ratio of different arboviral infections and the clinical aspects of infections with a particular emphasis on neuroinvasive diseases in this part of Europe (including Croatia) will be necessary to identify future trends and possible required interventions.

Identification of molecular mechanisms involved in the pathogenesis of neuroinvasive arboviral infections is a key step leading to the optimization of antiviral drug development in this field. Virus-induced modification of the biological environment of the infected cells that involves modification of nucleotides, carbohydrate, and lipid metabolic pathways can contribute to a better understanding of the neuropathogenesis associated with arboviral infections but also lead to the development of new therapeutic strategies that are a priority research area in this field [66].

## Figures and Tables

**Table 1:** Results of serology and RT-PCR testing in patients with neuroinvasive flavivirus infections.

Detected Virus	Serum Serology N (%) Positive		CSF Serology N (%) Positive		RT-qPCR N (%) Positive		Sequencing N (%)
	IgM	IgG	IgM	IgG	CSF	Urine	
TBEV (*n* = 11)	11 (100)	10 (90.9)	11 (100)	8 (72.7)	0 (0)	1 (9.1)	1 (9.1)
WNV (*n* = 32)	30 (93.7)	17 (53.1)	23 (71.8)	5 (15.6)	8 (25.0)	18 (56.2)	8 (25.0)

CSF = cerebrospinal fluid, TBEV = tick-borne encephalitis virus, WNV = West Nile virus.

**Table 2:** Virology results of cross-reactive serum samples of patients with neuroinvasive flavivirus infections.

Case	WNV IgM (Ratio)	WNV IgG (RU/mL)	TBEV IgM (Ratio)	TBEV IgG (RU/mL)	WNV IgG Avidity (AI)	VNT (Titer)	RT-PCR
1	Pos (4.1)	Pos (52)	Pos (1.5)	Pos (28)	16%	WNV 10	CSF Neg Urine Neg
2	Pos (3.6) Pos (3.3)	Pos (66) Pos (150)	Neg (0.7) NT	Pos (63) NT	16% NT	WNV 5 WNV 20	CSF Neg Urine Neg
3	Pos (4.0)	Pos (120)	Neg	Pos (69)	25%	WNV 10	CSF Neg Urine WNV Pos
4	Pos (5.3)	Borderline (19)	Pos (2.8)	Neg (6)	NA	NT	CSF WNV Pos Urine WNV Pos
5	Pos (4.4)	Pos (62)	Neg	Pos (31)	10%	NT	CSF Neg Urine WNV Pos

IgM ratio < 0.8 negative, 0.8–1.1 borderline, >1.1 positive; IgG relative units (RU)/mL < 16 negative, 16–22 borderline, >22 positive; WNV = West Nile virus, TBEV = tick-borne encephalitis virus, AI = avidity index < 40 low, 40–60 borderline, >60 high, CSF = cerebrospinal fluid, VNT = virus neutralization test, NA = not applicable, NT = not tested.

**Table 3:** Comparison of demographic and clinical characteristics in WNND and TBE patients.

Parameter	WNND (*n* = 32)	TBE (*n* = 11)			
	N (%)/Median (Range)		OR (95% CI)/U	*p*	Adjusted *p*
Sex (M)	18 (56.3%)	9 (81.8%)	0.29 (0.03–1.76)	0.166	0.374
Age (years)	65 (6–89)	36 (14–58)	289.5	<0.001	<0.001
Age group (years)					
<30	5 (15.6%)	2 (18.2%)	0.84 (0.11–10.24)	0.999	0.999
31–50	3 (9.4%)	7 (63.6%)	0.07 (0.01–0.42)	<0.001	<0.001
51–70	13 (40.6%)	2 (18.2%)	3.00 (0.50–33.08)	0.277	0.572
71+	11 (34.4%)	0 (0%)	NA	0.041	0.194
Day of disease	4 (1–14)	6 (2–15)	126	0.165	0.374
Hospital length (days)	12 (2–21)	9 (3–16)	195	0.384	0.700
Clinical diagnosis					
Meningitis	19 (59.4%)	7 (63.6%)	0.91 (0.16–4.54)	0.999	0.999
Meningoencephalitis	7 (21.9%)	3 (27.3%)	0.75 (0.13–5.63)	0.698	0.864
Symptoms (N)	3 (0–7)	4 (2–6)	119	0.111	0.333
Fever	38.7 (37.5–41.5)	39.0 (37.9–40.0)	121.5	0.480	0.712
Malaise	25 (78.1%)	11 (100%)	0.00 (0.00–1.92)	0.163	0.374
Headache	24 (75.0%)	11 (100%)	0.00 (0.00–1.57)	0.090	0.327
Nausea	16 (50.0%)	7 (63.6%)	0.58 (0.10–2.83)	0.501	0.712
Neck stiffness	15 (46.9%)	6 (54.6%)	0.74 (0.15–3.60)	0.736	0.864
Vomiting	11 (34.4%)	6 (54.6%)	0.45 (0.09–2.20)	0.300	0.527
Vertigo	6 (18.8%)	4 (36.4%)	0.41 (0.07–2.56)	0.248	0.515
Myalgia	6 (18.8%)	0 (0%)	NA	0.312	0.527
Photophobia	2 (6.3%)	3 (27.3%)	0.19 (0.01–1.92)	0.097	0.327
Arthralgia	3 (9.4%)	1 (9.1%)	1.03 (0.07–59.65)	0.999	0.999
Comorbidities (N)	1 (0–4)	0 (0–1)	286.5	<0.001	<0.001
Hypertension	18 (56.3%)	2 (18.2%)	5.56 (0.94–61.03)	0.039	0.194
Diabetes	10 (31.3%)	0 (0%)	NA	0.043	0.194
Abnormal neurological status	18 (56.3%)	5 (45.6%)	1.53 (0.32–7.79)	0.728	0.864
Abnormal EEG	30 (93.8%)	11 (100%)	0.00 (0.00–15.83)	0.999	0.999

WNND = West Nile neuroinvasive disease, TBE = tick-borne encephalitis, OR = odds ratio, CI = confidence interval, U = Mann–Whitney U test, NA = not applicable.

**Table 4:** Comparison of laboratory parameters in WNND and TBE patients.

Sample	Laboratory Parameters	WNND (*n* = 32)	TBE (*n* = 11)			
		Median (IQR)		U	*p*	Adjusted *p*
Blood	WBC count (×10^9^/L)	11.0 (8.2–12.3)	7.9 (7.5–11.6)	190	0.295	0.476
	% Lymphocytes	20.5 (10.0–25.0)	19.5 (16.3–21.0)	140	0.885	0.923
	% Neutrophils	69.5 (66.0–78.0)	69.5 (67.3–74.5)	141.5	0.923	0.923
	CRP (mg/L)	6.5 (2.9–13.0)	7.2 (3.8–15.2)	154	0.757	0.923
	ESR (mm/h)	15 (14–25)	22 (19–37)	6	0.155	0.465
CSF	Number of cells	88 (44–171)	131 (80–163)	133	0.295	0.476
	% of lymphocytes	51.5 (40.8–74.7)	90.0 (82.0–93.0)	68	0.008	0.036
	% of neutrophils	48.5 (25.3–58.5)	10.0 (7.0–18.0)	240	0.008	0.036
	Proteins (g/L)	0.71 (0.63–1.19)	0.68 (0.66–0.75)	199.5	0.317	0.476

IQR = interquartile range, WNND = West-Nile neuroinvasive disease, TBE = tick-borne encephalitis, NID = neuroinvasive disease, WBC = white blood cell, CSF = cerebrospinal fluid, CRP = C-reactive protein, ESR = erythrocyte sedimentation rate, U = Mann–Whitney U test.

**Table 5:** Binary logistic regression classification of WNND and TBE.

Predictor	Coefficient	Standard Error	*p* Value
Number of comorbidities	2.70	1.10	0.014
% of lymphocytes in CSF	−0.07	0.03	0.021

## Data Availability

The original contributions presented in the study are included in the article; further inquiries can be directed to the corresponding authors.
